# Effectiveness and safety of warm needle acupuncture for sciatica: A protocol for systematic review and meta-analysis

**DOI:** 10.1097/MD.0000000000024126

**Published:** 2021-01-15

**Authors:** Jing Zhang, Yujia Xie, Jia Li, Mingxing Yuan, Zongming Yu, Yue Su, Qianxiang Dai, Yuan Liu

**Affiliations:** School of Basic Medicine, Chengdu University of Traditional Chinese Medicine, Chengdu, Sichuan, China.

**Keywords:** meta-analysis, protocol, sciatica, systematic review, warm needle acupuncture

## Abstract

**Background:**

Sciatica is a syndrome characterized by sciatic nerve path and distribution area pain. Many kinds of literature reported the definite effect of warm needle acupuncture (WNA) on sciatica. However, there is no systematic review or meta-analysis on WNA for sciatica. Therefore, this study will design a protocol to comprehensively and systematically evaluate the effectiveness and safety of WNA on sciatica.

**Methods:**

The two researchers in this study will search the electronic database for randomized controlled trials, (RCTs) of WNA on sciatica, The databases we will search include PubMed, EMBASE, Cochrane Library, Web of Science, Chinese national knowledge infrastructure (CNKI), China Science and Technology Journal Database (VIP), Wanfang Database, and Chinese biomedical literature database (CBM). Review Manager 5.4 software and Cochrane risk bias tool will be us used for data analysis and to evaluate research quality. The main clinical indicators will include visual analogue scale (VAS) and Oswestry Disability Index (ODI).

**Results:**

This study will evaluate the efficacy and safety of WNA for sciatica.

**Conclusion:**

This study will provide a reliable scheme for objectively and comprehensively evaluating the efficacy and safety of WNA on sciatica.

**Trial registration number:**

INPLASY2020110074

## 1 Introduction

Sciatica is a kind of pain syndrome caused by compression and stimulation in the sciatic nerve pathway and distribution area. It is a common clinical peripheral nerve disease. Its typical symptoms include radiating pain in one or both lower limbs, sometimes accompanied by numbness, which can be manifested as pain in the waist, buttock, back of the thigh, posterolateral leg, and lateral dorsum of the foot. Sciatica may be sudden onset and followed by days or weeks.^[1,2]^ Sciatica is easy to develop into chronic and recurrent stage and can cause severe discomfort and functional limitation.^[3,4]^ Age-related degenerative disc disease compresses nerve roots and causes inflammation, which is an essential cause of sciatica.^[4,5]^ The pathogenesis of sciatica may be associated with the distortion of nerve roots or sensory ganglia, and may also be related to the role of local inflammatory cytokines.^[6]^ The prevalence of sciatica ranges from 1.2% to 43% among different studies.^[7]^ At present, the treatment of sciatica is a mainly conservative drug treatment and surgical therapy. However, conventional drugs for sciatica, such as non-steroidal anti-inflammatory drugs (NSAIDs), may provide short-term relief, and their long-term effects are uncertain.^[8]^ Furthermore, Surgical treatment is recommended only when the large disc breaks into the spinal canal, resulting in the bladder or intestinal sphincter failure.^[6,9]^ In general, these treatments of sciatica is limited and imperfect. Therefore, it is urgent to find a more effective and safe treatment for sciatica, which brings significant challenges to clinical practice.

Currently, as one of the most distinctive traditional Chinese medicine therapies, acupuncture has been widely used in clinical practice in China and many western countries.^[10]^ There are many clinical reports, systematic reviews and meta-analysis on acupuncture for sciatica.^[11–17]^ Among the numerous acupuncture therapies, warm needle acupuncture (WNA) can better combine the advantages of acupuncture and moxibustion, which has not only the analgesic effect of acupuncture but also has the warming effect of moxibustion Some studies have shown that WNA has better effects in relieving pain and promoting blood circulation compared with simple acupuncture or moxibustion.^[18,19]^ After searching the database, the authors found that there were many RCTs about WNA in the treatment of sciatica. These literature show that WNA may be more effective than ordinary acupuncture in the treatment of sciatica.^[20–33]^ Given the lack of systematic review and meta-analysis of WNA, the authors decided to design a protocol to provide evidence for a precise evaluation of the effect of warm acupuncture on sciatica and provide evidence for clinical application of WNA.

## 2 Methods

### 2.1 Protocol register

The protocol of our study has been registered on the INPLASY international prospective register of systematic reviews (INPLASY2020110074). Based on the systematic review and meta-analyses protocols (PRISMA-P) guidelines,^[34]^ we will conduct and report our program.

### 2.2 Eligibility criteria

#### 2.2.1 Types of studies

Only RCTs using WNA to relieve pain in patients with sciatica will be included. Retrospective studies, case reports, reviews, and other non RCTs studies will be excluded. For the included studies, their languages will be limited to English and Chinese.

#### 2.2.2 Types of participants

The patients to be included in this study should be those who have been diagnosed with sciatica or have been diagnosed with synonyms of sciatica, such as nerve root injury, nerve root compression and radiation pain under the knee, or have the following symptoms and signs: radiation pain of sciatic nerve distribution, Lasegue's sign, Kernig's sign, and Bonnet's sign. Patients’ age, gender, and nationality will not be restricted.

#### 2.2.3 The type of intervention

The studies we included in the experimental group using WNA alone or in combination with other commonly clinical treatments will be included. However, If it is not clear that WNA plays a role in the combination therapy, the combination of WNA with other therapies will be excluded. Conventional acupuncture, electroacupuncture or drug therapy will be used in the control group (such as NSAIDs).

#### 2.2.4 Types of outcome measures

##### 2.2.4.1 Primary outcome

The primary outcome is VAS and ODI.VAS can evaluate pain intensity by measuring continuous pain, and the data is converted into 0–100 mm. And the degree of daily dysfunction of patients can be evaluated by ODI. ODI includes pain, single function and individual comprehensive function, with ten items scoring 0–5 points for each item.^[35]^

##### 2.2.4.2 Secondary outcomes

The secondary outcome includes the 36-Items Short Form Health Survey (SF-36)^[36]^ (A quality of life assessment tool widely used in clinics), valuation criteria of Traditional Chinese Medicine (TCM) syndromes and adverse reactions caused by intervention treatment, such as fatigue, dizziness, etc.

### 2.3 Search methods for study identification

#### 2.3.1 Electronic searches

The following eight databases will be used for searching controlled clinical trials, including PubMed, Web of Science, Cochrane Library, EMBASE, CNKI, CBM, VIP, and Wanfang Database. The retrieval period will start from the establishment of each database to November 2020. We will use “RCT, sciatica, WNA” as our search terms. Table 1 provides a detailed search strategy in PubMed, search terms, and search strategies with the same meaning will be similarly used in other databases.

**Table 1:** Search strategy used in PubMed database.

Number	Search items
1	randomized controlled trial.pt
2	controlled clinical trial.pt
3	randomized.ti,ab
4	randomly.ti,ab
5	groups.ti,ab
6	trial.ti,ab
7	or 1–6
8	sciatica.ti,ab
9	sciatic neuralgia.ti,ab
10	ischialgia.ti,ab
11	ischioneuralgia.ti,ab
12	discogenic sciatica.ti,ab
13	bilateral sciatica.ti,ab
14	disc herniation-inducedsciatica.ti,ab
15	or 8–14
16	Acupuncture.ti,ab
17	warm acupunctrue.ti,ab
18	warm needle.ti,ab
19	warm acupuncture therapy.ti,ab
20	moxibustion.ti,ab
21	warm needling moxibustion.ti,ab
22	needle warming moxibustion.ti,ab
23	warming-needle moxibustion.ti,ab
24	Needling.ti,ab
25	Acupoint.ti,ab
26	or 16–25
27	7 and 15 and 26

#### 2.3.2 Additional search

Meanwhile, we also plan to search other relevant literature, such as grey literature, meeting minutes, and all reference lists of included studies. The retrieval of this literature will not be limited by date, country, publication status, or year of publication.

### 2.4 Data collection and analysis

#### 2.4.1 Collection of data

**Figure 1:**
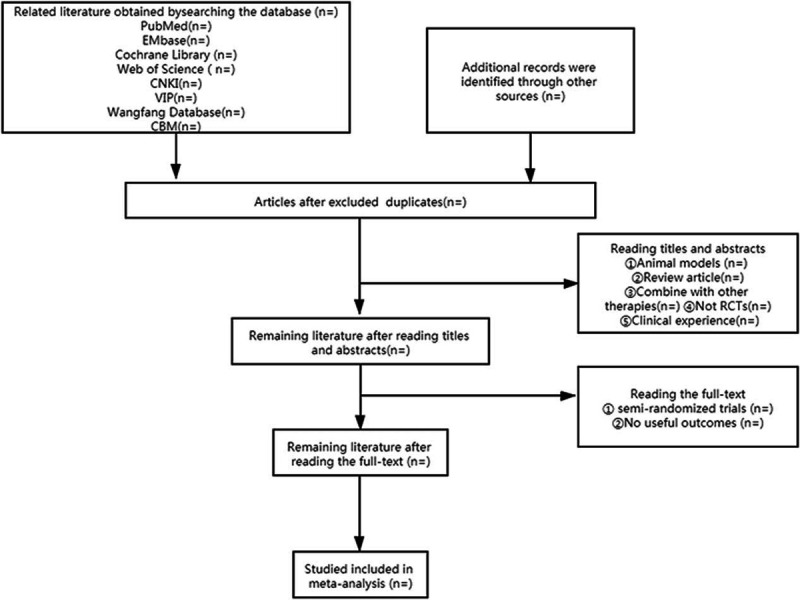
Flow chart of the literature screening and selecting process.

According to our previous search strategy, the retrieved literature will be imported into EndnoteX9 by two independent researchers. After removing the duplication through software, they will evaluate the title and abstract of the study based on previously established criteria. After assessing, the retained literature will be carefully read and re-selected. Any divergences between the two reviewers will be reached through third-party evaluation or group discussion. According to PRISMA guidelines,^[37]^ we developed selection procedures. The selection flow chart is shown in Figure 1 (Fig. 1).

#### 2.4.2 Extraction and management of data

Structured, standardized forms will be used by two researchers to collect research data independently. A third person or panel discussion will resolve any differences between the two investigators on data collection. If there is unclear or incomplete information, we will approach the lead author of the article by email or phone for details. The extracted information will include:

(1) Necessary information (e.g., author, year of publication)
(2) Interventions in the control and treatment groups
(3) Necessary information (e.g., number of participants, gender, average age, etc.)
(4) Outcome indicators
(5) Randomized methods

### 2.5 Quality assessment of the included literature

Cochrane Collaboration Tool will be used to assess included studies. This will be done by two authors independently assessing the quality of the methodology, which covers seven aspects: random sequence generation, allocation concealment, blinding of participants and personnel, blinding of outcome assessment, incomplete outcome data, selective reporting, and other biases. After evaluation, the literature quality will be divided into “high”, “low” or “unclear”.Disputes arising from an evaluation process are discussed and resolved with the fourth author (MY).

### 2.6 Data analysis

#### 2.6.1 Statistical analysis

Statistical management software (RevMan V5.4) will be used for data processing and meta-analysis. Mean difference (MD) and rate ratio (RR) will be used to evaluate continuous variables and dichotomous variables in extracted data respectively. Meanwhile, 95% confidence intervals (CIs) is also being used to evaluate these two variables.

#### 2.6.2 Heterogeneity assessment

We will use I2 values according to the Cochrane Manual to assess heterogeneity and analyze valid data based on the evaluation results. If I^2^ > 50%, it shows that the heterogeneity among the studies is significant, we will select the random effect model analysis and conduct further subgroup analysis. When I^2^ < 50%, the heterogeneity between studies is small, and the fixed effect model will be used by us.

#### 2.6.3 Subgroup analysis

If the included data are highly heterogeneous, we will perform subgroup analysis according to sample size, study quality, and interventions.

#### 2.6.4 Sensitivity analysis

If the heterogeneity between the included studies is high, we will conduct a sensitivity analysis. If the sensitivity analysis does not substantially change the results, it indicates that the results of the study are credible; if the sensitivity analysis draws different conclusions, it suggests that there are potentially important factors influencing the effectiveness of the intervention.

#### 2.6.5 Assessment of publication bias

If we include more than ten studies, we will consider using Revman5.4 software to plot funnel plots for publication bias analysis.

### 2.7 Ethics and dissemination

No ethical approval is required for this study. The included data are from published literature,

This study provides a comprehensive evaluation of the efficacy and safety of WNA in the treatment of sciatica and will be published in peer-reviewed journals or conferences.

## 3 Discussion

Sciatica is one of the common clinical diseases, which is easy to recur, significantly reducing the work efficiency and quality of life of patients. However, whether it is drug therapy or surgical treatment, the efficacy is not very satisfactory. Studies have shown that warm acupuncture can combine the effects of acupuncture and moxibustion. Acupuncture can stimulate nerve transmission, improve pain threshold when it is used with moxa sticks, it can penetrate the skin, direct heat to muscular layer and nerve, strengthen local blood circulation, promote the resolution of neuroedema and inflammation, and promote nerve repair.^[38]^ Other studies have shown that warm acupuncture can inhibit inflammation, reduce pain degree and improve clinical symptoms by increasing pain threshold^[21]^ and decreasing serum levels of PGE2, IL-6^[22]^ and plasma levels of 5-HT, SP, TXB2, and T/K ratio.^[24]^

From what has been reported, this study will be the first systematic review and meta-analysis of WNA for sciatica, and our results will provide objective statistical data for WNA for sciatica, provide a reliable reference for clinicians in the treatment of WNA sciatica, and make it more widely available to the public.

## Author contributions

**Conceptualization:** Jing Zhang, Qianxiang Dai.

**Methodology:** Mingxing Yuan.

**Resources:** Yujia Xie, Jia Li.

**Software:** Zongming Yu, Yue Su.

**Writing – original draft:** Jing Zhang, Yuan Liu.

## Abbreviations

Abbreviations: ODI = Oswestry Disability Index, RCTs = randomized controlled trials, VAS = visual analogue scale, WNA = warm needle acupuncture.
